# Developmental disability among Aboriginal children in Western Australia (WA).

**DOI:** 10.23889/ijpds.v7i3.1855

**Published:** 2022-08-25

**Authors:** Alison Gibberd, Sandra Eades, Nicoletta Psilos, Jenny Bourke, Helen Leonard, Emma Carlin, Anne Kavanagh, Roz Walker, Melissa O'Donnell, Rebecca Colbung, Lesley Nelson

**Affiliations:** 1 The University of Melbourne; 2 Telethon Kids Institute; 3 University of Western Australia; 4 University of South Australia; 5 South West Aboriginal Medical Service

## Objectives

While Aboriginal Australians are known to be disproportionately affected by intellectual disabilities (ID) and/or autism spectrum disorders (developmental disabilities), true prevalences among Aboriginal children are unclear, with evidence of delayed and missed diagnoses and barriers to services. This study estimated these prevalences and disability service use among WA Aboriginal children.

## Approach

Two cohorts and data sources were used. Firstly, a state-based health and disability data linkage for all WA Aboriginal children born 2000-2013, including hospital, public outpatient mental health, birth and death data and state registries of birth defects, cerebral palsy and ID. The cumulative incidence of diagnosis by age 18 was estimated. Secondly, prevalence of service access was estimated from all National Disability Insurance Scheme (NDIS) WA Aboriginal and non-Aboriginal participants aged 0-17 on 30/6/2021 with a primary diagnosis of ID (with or without fetal alcohol spectrum disorder (FASD)) or autism. Census population counts were denominators.

## Results

Using the data linkage, 3.3% of Aboriginal children born 2000-2013 were diagnosed with developmental disability by 18 years. The most common diagnosis (2.5%) was ID without FASD. Using NDIS data, 3.1% of Aboriginal children were NDIS participants with development disability in 2021, but only 0.7% of children with ID without FASD. Instead, autism was most common diagnosis. Aboriginal children were more likely than non-Aboriginal children to be NDIS participants with autism (prevalence ratio (PR): 1.16, 95% confidence interval (CI): 1.06-1.26), ID without FASD (PR: 2.06, 95% CI: 1.80-2.35) and ID with FASD (PR: 40.5, 95%CI: 29.9-57.1). For both data sources, prevalences differed by region. Aboriginal NDIS participants with ID had an older age distribution than non-Aboriginal participants.

## Conclusion

For both cohorts, the prevalence of developmental disability was >3%, though the contributions of ID and autism differed in the two data sources. Variation in diagnoses by region and Aboriginal status may indicate differential diagnosis and variation in age suggests delayed diagnoses or access to services for Aboriginal children.

